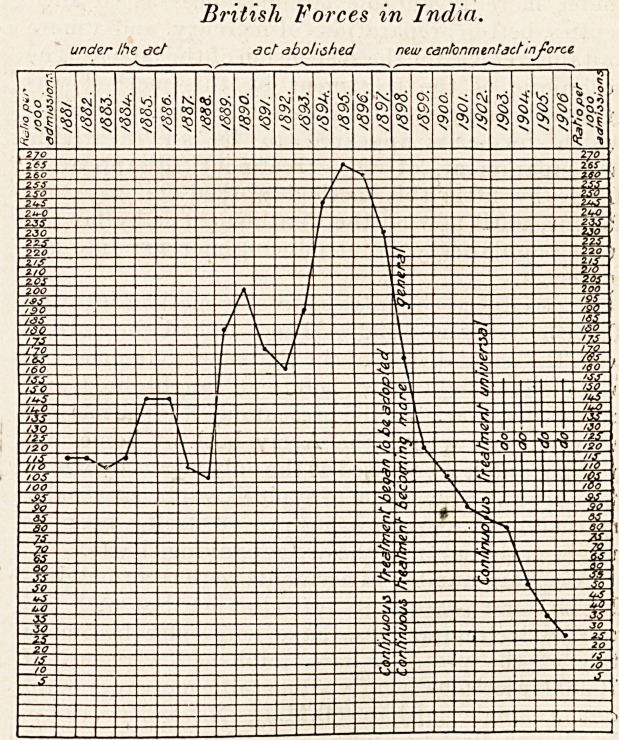# The Present Day Treatment of Syphilis in England

**Published:** 1907-06-29

**Authors:** F. J. Lambkin

**Affiliations:** O.C., Military Hospital, Rochester Row, London, Lecturer in Syphilology at the Royal Army Medical College


### Hospital Clinics.

### THE PRESENT DAY TREATMENT OF SYPHILIS IN ENGLAND.

By COLONEL F. J. LAMBKIN, R.A.M.C., O.C. Military Hospital, Rochester Row, London, Lecturer in Syphilology at the Royal Army Medical College.

During the last twenty years the treatment of syphilis has made much progress on the Continent of Europe, and in the British Army both at home and abroad. Does this apply equally among the civil population of the United Kingdom? This will be the subject for consideration in the following paper.

It is convenient to divide the subject of the treatment of syphilis into three parts:—(1) Hygiene (including the important question of the increase and maintenance of tissue metabolism); (2) the administration of the specific, mercury; (3) the employment of auxiliary measures, such as iodide of potash, etc.

The consideration of all these elements opens up a very large question, too large for the scope of this paper, hence it is proposed to limit discussion to the second—*i.e.* the modes of administration of mercury. Before discussing this, it will be necessary to inquire what the teaching is in England to-day as to the actual practical treatment of the disease. This can best be studied at the out-patient department of any of our large hospitals, as it is seldom that patients suffering from syphilis, plain and simple, are admitted as in-patients to any of these institutions. We are taught there to treat the symptoms and lesions of it which may be present, but too often on the disappearance of these he ceases to come to hospital; consequently treatment is suspended, and is not resumed until fresh manifestations render it necessary. Should these latter be so mild as to cause little inconvenience or disfigurement, he probably receives no further treatment, as he may not present himself until he is affected with something more serious in the shape of, say, cerebral or spinal paresis. The fact is that the treatment of syphilis is aimed more at obliteration of apparent symptoms than at an actual cure. This may not be the teaching as expounded at the medical schools, but it is what one sometimes sees in practice at their clinics. What we learn at the hospitals in England is to ameliorate and not to cure the disease. It is an easy matter to disperse the symptoms and lesions of syphilis. For as soon as their cause is diagnosed, all that is necessary is to apply that remedy which we know to trust so well—mercury—and they disappear. But it is entirely a different thing to treat the disease with a view to eventual cure or prevention of untoward symptoms.

But in the face of what we now know to be necessary as regards the treatment of syphilis, and on which all syphilologists of the day are agreed—*i.e.*, that to cure the disease and to prevent its remote effects upon the patient or his posterity—it is absolutely necessary that treatment should be carried out over a lengthened period, with a minimum of 18 months, by some plan or other, be it continuous, chronic-intermittent, or preventive.

Further, most authorities are agreed that syphilis needs to be attacked not only during its eruptions, but also in its quiescence; hence whatever system of treatment is established, it is necessary that it should be of long duration. It should always be remembered that syphilis is a chronic disease or diathesis requiring chronic treatment. It is not on such lines as these that the system of treatment which we see carried out at our great hospitals is founded. On the contrary, what exists there not only makes no attempt at curing, but what is possibly still worse, does not even prevent further and permanent trouble.

In the way of argument, I have heard it stated over and over again that what we see of the treatment of syphilis at a hospital is not to be taken as an example of how the disease is treated throughout the country generally, and that quite a different state of affairs exists in private clinical practice. A unique experience on both sides of the question unfortunately makes me think otherwise, and forces me to the conclusion that as regards the treatment of syphilis in civil life in the United Kingdom there is little difference in the way it is carried out between that in vogue in the hospitals and in private practice. After all, "as the twig is bent, so will it grow," and the student who has been accustomed to see the disease treated at the hospital in a certain manner will be at least prone to continue the same method when he enters private practice. From my experience I unhesitatingly say that in 99 cases out of a hundred in England at the present day the system of treatment which is carried out is much what we see it is at our large hospitals, as above described. In other words—and there is no good our blinking the fact—the treatment of syphilis in England at the present time is one of symptomatic amelioration, and, generally speaking, no real attempt is made to deal with the disease in the only possible way of doing so, if any hope is to be held out of an eventual cure, *i.e.* of establishing some system by which treatment can be carried out with as little doubt as possible over that lengthened period which we know to be necessary, and in this respect the conclusion I have come to is that at the present day the treatment of syphilis in the United Kingdom among the civil population has not kept pace with what has been done in this respect during the last 20 years on the Continent and in the British Army. This conclusion I have arrived at from what I have personally seen of the treatment of the disease in civil life at the clinics of the largest hospitals in England, as compared with that adopted generally in Germany, France, Italy, and at the syphilitic clinics of Paris, Berlin, and Milan, added to my knowledge of the advance the treatment of the disease has made throughout the British Army in all parts of the world.

The question is what are the causes or factors which have probably or possibly led to this state of things. Personally, I do not think one requires to seek far to find them. To my mind, the following are the principal causes:—(1) Neglect to bring syphilis within the Infectious Diseases Notification Act; (2) the non-adoption in England of the more modern method of administering specific treatment. There are other possible causes of minor consequence. As regards the first of the two main causes, it was a very great mistake that syphilis was not included in the Infectious Diseases Act. I have always thought that the medical profession in England were responsible for this omission. They should have insisted upon this point. One Royal Commission has strongly recommended the inclusion of syphilis on the Notification of Diseases Act, but the profession has not supported its recommendation.

One of the chief causes which has been put forward by the profession for not carrying the treatment over a lengthened period is that the uneducated classes in England cannot be made to see the necessity of further treatment after the disappearance of the symptoms of the disease. This is the reason why syphilis should be included in the Act. Patients would then be eager to get cured, and if they failed to present themselves for treatment would be compelled to do so. The insuring of better treatment means decrease in the amount of disease—lunacy, general paralysis, tabes, and other diseases due to syphilis—but it would also act as a real check on the spread of the disease, and probably ultimately stamp it out. We are told that eventually education will do for the treatment of syphilis everything for which this Act is proposed, without its enactment; but I fear we should have to wait many a year before seeing education reach this mark in England.

When the old C.D. Acts required upholding the medical profession entered into the fight with all its energy, and brought all its great influence to bear on the matter; yet this Act was one of pure prevention, whereas the Notification Act aims also at cure. The question at issue is one of political economy, one affecting in the most serious manner the health of the nation, and as such is surely worthy of the gravest attention of the medical profession. We cannot help feeling convinced that the fact of making syphilis a notifiable disease would be of infinite value to the nation at large.

In considering the second cause—*i.e.*, the non-adoption of the more modern modes of administering specific treatment—I am aware that I am treading upon tender ground. I shall probably be told that I am advocating one particular method because I am prejudiced in its favour, or that the reason why these methods have not been adopted is because experience in England is against their employment; but the question at issue is not one of a comparison as to the relative advantages of one method over another as regards their respective therapeutic effects on syphilis, but it is one the essence of which is, (1) Which of all the known methods of administering mercury will enable us best to continue treatment over that length of time which we know to be absolutely necessary to effect a cure of syphilis, and in any case act as a preventive of its near and remote consequences? (2) By which of the known methods can we do this with the greatest certainty, and regularity, and with the least inconvenience to our patients?

It will suffice here to mention the three principal modes by which mercury is introduced into the system:—(1) The internal, ingestive, or stomachic method; (2) the inunction method; (3) the intramuscular injection method (in speaking of which I mean always that of the insoluble salts of mercury). The internal method is the one which is generally employed in England—in fact, it is the favourite method in at least 90 per cent. of the cases under treatment. By this mode of medication mercury is introduced as pills or powders, mixtures, or syrups for absorption either by the stomach or intestines. The technique of this particular method has varied but little as years have gone on, except in the form of the salt of the metal which has been employed. At the present day the most popular forms are the original salts—namely, bichloride, and protoiodide, calomel, and metallic mercury. Each has its own admirers, and each is favoured in one country more than another; bichloride and protoiodide being generally used on the Continent, whilst on the whole metallic mercury is most favoured in England. Of late years calomel has become more or less discredited, owing to certain accidents which followed its use.

In England metallic mercury, being most favoured, is generally given in the form of a pill or powder, one of which is ordered to be taken two, three, or four times a day, as the case may be. Bichloride, when employed, is given in mixture or syrup. In this case it is very often combined with iodide of potassium, and forms a staple mixture to be found in almost every dispensary in the country. On the Continent bichloride and protoiodide are the favourite salts, and as such are given in the form of pills under the name of Dupuytren's or some modification of them—one pill to be taken from two to four times a day. The average dose per day of the bichloride is one-half grain for a man, one-third grain for a woman. France, especially Paris, is the home of protoiodide, where it is a general favourite. It is given in pills two to four times a day, with an average daily dose for an adult (male) of 1½ grains; half of that dose for a woman. Both of these salts have their admirers and adherers who claim for them certain advantages.

The following appears to be the general consensus of opinion concerning them. Protoiodide is better tolerated than bichloride, hence its dose can be raised with greater safety than in the case of the latter: it affects the gums sooner, but is less likely to salivate than bichloride; it is more likely to affect the intestine than the stomach, and often brings on diarrhœa. It benefits early secondary symptoms more than later lesions. Bichloride is not so well tolerated as the former salt, and it becomes dangerous to raise its dose above 1 grain per day. It affects the stomach rather than the intestines, and is very apt to cause gastritis, and also a painful condition called sublimate gastralgia. It ought not to be continued for longer than a few weeks at a time on account of its known effects on the stomach. Bichloride has most effect in the later secondary and tertiary stages. On the whole, although nothing definite is settled, preference is generally given to the protoiodide.

These are the chief ways by which mercury is administered by the ingestion method, and the salts described are those usually employed; besides them, it will be only needful to mention here a few of the principal modern salts of mercury which have during the last few years been introduced with the supposed advantage of being free from some of the disadvantages of the older salts, such as not causing stomatitis, salivation, diarrhœa, or gastritis. The following are the salts:—Tannate, salicylate, and sozoiodol of mercury. I do not believe that any one of these has proved itself better than the older salts, on which most practitioners have to fall back.

The next method of administering mercury is that by inunction. This is the oldest method of all, but has never been used in England in such a general way as the one just described. It was in full use with us in the fifteenth century, and John Hunter, in speaking of the ways of introducing mercury into the system, says:—"When it can be thrown into the constitution with propriety by the external (inunction) method, it is preferable to the internal, as the skin is not nearly so essential to life as the stomach." It is probable that had more attention been paid and importance attached to the details of this method in England, it would still be flourishing. As its entire success depends upon the manner in which this is done, and as it has been almost entirely ignored in this country, of late years this special form of treatment has not held its own except in a few institutions. The Mecca of the inunction method is Aachen, where it has flourished for the last century and a half, and where it is done to perfection.

At the Military Hospital, Rochester Row, London, the external method is carried out as follows:—(1) First a rubber is necessary, and for this purpose orderlies are educated; (2) before inunction is performed a hot bath is given for 20 minutes; (3) the following formula is used for the mercurial preparation:—

| | |
|---|---|
| Ung. hydrarg. ... ... ... ... | grs. 50 |
| Lanolin. hydros. ... ... ... | grs. 25 |
| Adipis benzoin. ... ... ... ... | grs. 25 |

Divide into two parts and wrap in wax paper.

Both parts are handed to the rubber. The regions of the body rubbed are changed daily so as to avoid the effects of friction. They are, in succession:—First day, arms; second, forearms; third, thighs; fourth, calves; fifth, back; sixth, chest. Each rubbing lasts from 20 to 25 minutes. It must be done slowly, evenly, with a good deal of pressure. The part, after being rubbed, if properly done, ought to look as if black-lead had been used—shiny, but not greasy. The best time for inunction is in the morning, as the movements caused by exercise favour absorption. The part rubbed on any one day is not scrubbed until the morning prior to its being again utilised. During the course of rubbing the usual strict attention is paid to the hygiene of the mouth and gums, and close watch kept on the condition of the urine and body-weight. A course consists of 40 rubbings. How different is this procedure from that which one sees taught and carried out in this country, where we learn that to do it correctly it is sufficient to tell the patient to take a piece of mercurial ointment the size of a hazel nut on the top joint of the index finger and to rub this into the groin or calf of the leg before going to bed. That sort of inunction fails, and is dropped.

There are grave objections to this method. First, it is difficult to carry out efficiently, except in a

*United Kingdom.*

*British Forces in India.*

hospital. Second, it is dirty, repugnant and compromising, as it stains linen, and servants and washerwomen soon get to know what is going on. A third objection arises from its effects—namely, stomatitis and mercurial dermatitis of a severe nature being often associated with it.

### The Intra-muscular Injection.

The third method of administering mercury is by intra-muscular injection. This was first introduced by Scaranzio, of Pavia, in 1864, but although therapeutically a success, it had to be abandoned owing to the number of accidents that accompanied it, such as abscesses. The accidents were undoubtedly due to the absence of antiseptics at that period. The method was reintroduced by Smernoff in 1882, with very little better success, and was again dropped. It was finally brought forward in 1887 by Balzar, under more favourable circumstances as to antiseptics, better preparations of mercury, and a more suitable vehicle for the suspension of the latter. The technique of the treatment by this method was gradually improved until accidents, such as abscess, which formerly appeared to be an invariable accompaniment of the treatment, disappeared. Little by little this method worked its way into favour, until now on the Continent it is by far the most popular way of introducing mercury into the system.

I introduced the intra-muscular method into the British Army in 1890.[1] There it had to contend with the usual dislike of the Britisher to innovation, and had to fight hard before it caught hold and became popular. At the present day it is the method adopted throughout the Army both at home and abroad, and is recognised as the only means by which the cure and prevention of syphilis can be effectually carried out. As such it has proved itself a boon, as can be gathered from the accompanying charts.

A certain number of accidents were recorded during the first years of its introduction, but with improved technique these have long since become a tale of the past. I have published a record of 70,000 injections (nearly all metallic mercury) without a single mishap (B.M.J., November 1905). During the last two years at the Military Hospital, Rochester Row, London, no fewer than 11,000 intramuscular injections have been given, with a total absence of any accident. I have no intention here of entering into the technique of the intra-muscular method. Suffice it to say that in the Army the insoluble salts of mercury are for several reasons preferred to the soluble, and of these metallic mercury is the form which is generally used. The insoluble salts need be injected only once a week, whereas if the soluble salts are used a daily injection is required.

### The Plan Adopted.

On the syphilitic patient becoming sick, he is placed on a syphilis register, and provided with a case sheet upon which is entered his previous history and present state. Whenever he attends the treatment is entered on his sheet. A course of six weeks' energetic treatment, usually in hospital, is given, which involves six mercurial injections. On finishing this course the patient is allowed an interval of two months without any treatment, but during that time is inspected once a fortnight. Should he remain free from syphilitic manifestations for two months he is then ordered a further course of four injections once a fortnight. If fresh symptoms appear, a second course of six injections weekly are given, followed by a two months' interval. If free from signs of the disease, the next interval is increased to four months, followed by a course of four injections. The succeeding interval may be increased to six months, followed by four injections, one each month.

In a tabular form the above reads:—

Six weeks' treatment: Six mercurial injections. Two months' interval.

Two months' treatment: Four injections. Four months' interval.

Two months' treatment: Four injections. Six months' interval.

Four months' treatment: Four injections.

Total, 21½ months' treatment.

The above will only apply to patients who have had no further relapse. For those who have relapse, treatment must be extended very much longer.

Thus it will be seen that syphilis is treated in the Army not only with the view of relieving its present manifestations, but of also preventing further trouble and bringing about a cure of the disease itself. There is no compulsion as to the actual method which is to be employed, but there is as regards continuous treatment; and as medical officers have long since discovered that the only possible practical way of carrying this out is by intramuscular medication, they have universally adopted it in preference to all others.

In comparing these three methods of administering mercury, it must suffice here to do so from the point of view of convenience, rather than from that of therapeutics. For even granted that they are all three equal as regards the latter, what we have to see is, which of them is the most convenient method, both for the patient and surgeon, and which of them will enable us best to carry out that prolonged treatment which we know to be absolutely necessary to procure prevention and cure. To take the ingestion method first, what are its supposed advantages as regards convenience? It is claimed for it that it is practical, that it is easy and convenient, as well as certain in its merits. In fact, what is more simple than to swallow every day one or two pills, or one or two spoonfuls of any kind of mercurial preparation? As regards its being "practical," on the ground that it is easy and convenient, this may possibly be true as compared with treatment by inunction; but it is certainly not when brought side by side with the intra-muscular method, which calls for only one injection a week. It is decidedly not nearly so "certain" a line of treatment as either inunction or injection, for the amount of mercury which is absorbed into the system is uncertain, owing to the irregularity with which patients take their powder, pills, or medicine.

[1] See "The Treatment of Venereal Disease and Scabies in the Army, 1904." Advisory Board of Army Medical Services, First Report, 1904, page 28.

This among the lower classes is the result of deliberate neglect, whilst in the upper and educated classes it is nearly always the result of forgetfulness. As regards "simplicity," ingestion compares favourably in this respect with inunction, but certainly not with injection of the insoluble salts of mercury, when, instead of having to take pills, powder, or mixture four times a day over extended periods, all that is needed is that the patient pay a visit to his doctor on one day a week or fortnight, and is not detained for longer than a few minutes.

As regards the advantages of the inunction mode of treatment, I think that as anything like a routine measure it cannot be recommended.

The advantages of the intra-muscular method are:—(1) Treatment is in the hands of the medical man, who is thus certain as to whether the patient gets treated or not; (2) definite dosage with almost certain absorption; (3) treatment calls upon the patient's time for only a few minutes once a week; (4) cleanliness and professional secrecy; (5) non-interference with the digestive system, which it lets free to do other work.

The disadvantages are:—(1) Pain, nodosities, and abscesses occurring at site of injection; (2) mercurial stases. As regards pain: with improved technique is has practically disappeared. As a rule there is absolutely none, and, if present, is of such an infinitesimal nature as not to be worth notice. The same may be said as regard nodosities and abscesses, to say nothing of the much-vaunted emboli. To my mind, the only legitimate objection that can be raised against the intra-muscular method is that, once the injection is given, there the mercury must remain until absorption takes place, so that should stomatitis intervene the cause of it cannot well be removed. This has been reported to have resulted in grave salivation, a state of affairs it has never been my lot to see. Otherwise, I cannot conceive one argument in favour of the ingestion or inunction methods of treatment over the intra-muscular. On the contrary, as a convenient plan, everything favours the latter, and personally, if I had to choose between these three methods as to which I should select to be treated by, I should not hesitate to plump for the intra-muscular. I should say to myself, if I select the internal I shall be obliged to swallow medicine in some form or other two, three, or four times a day for months, and to be reminded each time of the skeleton in my cupboard, and am certain sooner or later to suffer from diarrhœa, gastritis, or enteritis, when treatment will have to cease, and I will be left in a worse plight than before. Or should I select inunction, I shall have to undergo a rubbing each day, which will take me, all told, the best part of an hour; whereas if I choose the intra-muscular, all the trouble or inconvenience I am put to is a visit of a few minutes once a week or once a fortnight to my doctor. I may possibly feel a small amount of pain or stiffness after each visit, but I need not think any more of treatment until the next attendance. To my mind, we have in the intra-muscular method a plan of treating syphilis which places us in a position not only to ameliorate present symptoms, but also to prevent future ravages, and eventually effect a cure of the disease. Why is it, then, that this which has proved such a boon on the Continent of Europe and in the British Army has not been universally adopted in England? I cannot help thinking that one of the chief reasons for this is conservatism on the part of the profession, or, in other words, its rooted dislike to anything new. Although born in England, antiseptic surgery took longer to spread there than in any other country; in the same way, although the intra-muscular method was first introduced by an Englishman—Berkeley Hill—in a paper which was published in *The Lancet* in 1854, it went unnoticed and ignored by his countrymen.

In a French work before me there is an epitome of the various medical opinions which were held in different countries in the year 1882-83 as regards this method, of which most authorities even then spoke enthusiastically as to its therapeutical effects. I find the following as regards England:—"En Belgique et en Angleterre, on ne se préoccupe pas beaucoup de cette méthode. And thus it is to-day, with the result that treatment of syphilis in England is one of symptomatic amelioration rather than any real attempt at prevention or cure.

The success which has followed the plan of treating syphilis in the Army is an object lesson, and until some such like method is adopted generally throughout England things are likely to remain as they are, and syphilis will be inefficiently treated.

## Figures and Tables

**Figure f1:**
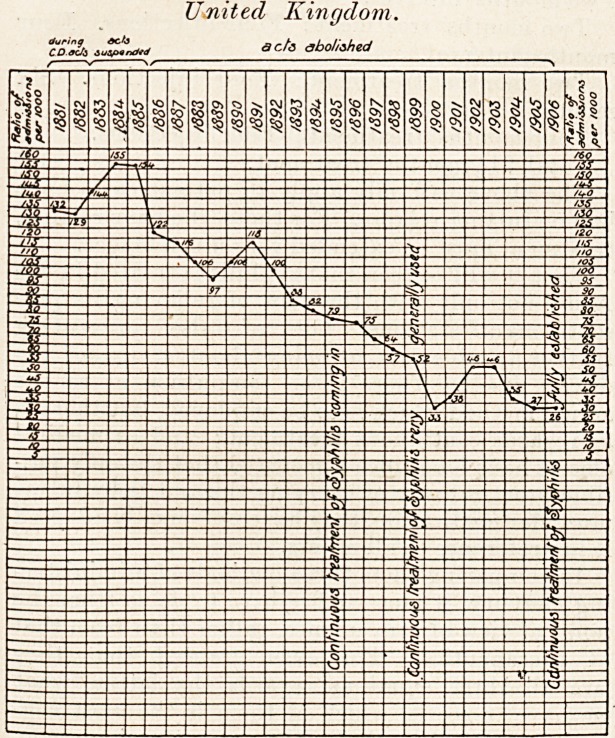


**Figure f2:**